# Can digital economy promote urban export sophistication? Evidence from China

**DOI:** 10.1371/journal.pone.0308285

**Published:** 2024-11-01

**Authors:** Qin Zhu, Haijing Yu, Zhimei Wan

**Affiliations:** 1 School of economics, Zhejiang Gongshang University, Hangzhou, P.R. China; 2 Business School, Xiangtan University, Xiangtan, Hunan, P.R. China; Leshan Normal University, CHINA

## Abstract

This study investigates the impact of digital economy development on urban export sophistication and its mechanisms. We use the chain mediation effect model to analyze the panel data of 281 cities in China from 2011 to 2017. The results show that the digital economy has a significant and positive impact on urban ES. There are two main influence paths. One promotes urban ES by accumulating human capital; the other stimulates technological innovation and further leads to urban ES. The “technological innovation effect” plays the most considerable mediating role among them. The heterogeneity test result shows that the digital economy impact is greater in western China than in eastern and central regions. We further show that the influence of the regional digital economy on urban ES presents an inverted U-shaped curve. Our paper provides guidance for promoting the construction of and the high-quality development of trade.

## Introduction

Against the backdrop of increased uncertainty in the global trade environment, cultivating competitive trade advantages and thus promoting high-quality trade development has become an issue of great concern to all countries [1, 2]. Among them, as the world’s largest trade exporter, China’s total export trade reached USD 3.36 trillion in 2021, accounting for 15% of the world’s share. [Data source: World Trade Report 2021 https://www.wto-ilibrary.org/content/books/9789287051400] With the continuous expansion of trade scale, the future development direction of China’s trade is to improve the technical complexity of export products, promote the upgrading of manufacturing industry to medium and high-end, and realize the transformation from a large trading country to a powerful trading country. Export sophistication (ES) is widely used to measure the level of trade development and can reflect whether the commodities structure is optimized and positively correlates with regional economic growth [3]. ES is also related to the trade balance. A country can fall into the middle-income trap if its ES cannot be effectively upgraded [4]. Therefore, in-depth exploration of the factors that affect the complexity of export technology has significant theoretical and practical significance for formulating and implementing national export strategies.

In recent years, scholars have thoroughly explored the influencing factors of export complexity (ES) from multiple perspectives. For example, Su et al. (2020) studied the impact of service trade restrictions on the export complexity of manufacturing industries. They found that the opening up of service trade significantly increased the export complexity of these industries [5]. Song et al. (2022) analysed the impact of intermediate goods imports and independent innovation on the export complexity of Chinese manufacturing firms. They found that independent innovation played a key role in enhancing export complexity [6]. In addition, Li et al. (2021) investigated the impact of foreign direct investment (FDI) on the export complexity of Chinese manufacturing industries. Their results showed that FDI had a significant positive impact on export complexity [7]. These studies indicate that various factors, such as market entry mode, location factors, and financial markets, have important impacts on ES.

However, with the rapid development of the digital economy, its impact on export sophistication (ES) has not been fully studied. The digital economy, especially the wide application of information and communication technologies (ICT) and internet platforms, is reshaping the international trade landscape. Nath and Liu (2017) studied the impact of ICT on services trade. Their results showed that the application of ICT significantly promoted the development of services trade [8]. Liu et al. (2024) explored the role of digital technologies in the restructuring of global value chains. They pointed out that the application of digital technology helps to enhance export complexity [9]. At the micro level, Deng et al. (2022) examined the impact of digital platforms on exporters’ rapid internationalisation and exit. They confirmed that the application of digital platforms significantly boosts export growth [10]. While, Mu et al. (2020) show that the main impact of the Internet on exports is that the use of Internet platforms reduces the information asymmetry between producers and consumers and improves the efficiency of transactions, which in turn has a profound effect on international trade [11]. These studies suggest that the digital economy may be a key factor influencing ES.

On the other hand, China’s digital economy driven by digital technology is developing rapidly at the moment, with the scale of the digital economy growing from 16.2 trillion in 2014 to 39.2 trillion in 2020 [Data source: Digital economy development in China (2021) http://www.caict.ac.cn/kxyj/qwfb/bps/202007/P020200703318256637020.pdf], with an average annual growth rate of 15.87%, and the scale of cross-border e-commerce reaching 6 trillion yuan in the same year. However, few studies on the ES from a digital economy perspective have been conducted, especially in the context of China, where both the digital economy and export sectors have seen significant development. Does the development of the digital economy affect ES, and, if so, what are the underlying mechanisms? The study of these issues is of great practical importance for improving the position of a region in the global value chain and optimizing the trade structure.

To fill this gap, this paper explores the impact of the digital economy on the urban ES and the mechanisms underlying this impact. The contribution of this paper is threefold. First, we measured the level of urban digital economy development by constructing a system of indicators. We used it to study the impact of the digital economy on the urban ES. To the best of our knowledge, few scholars have covered this yet. Interestingly, our study not only reveals a positive effect between the two, but also finds a threshold effect, which appears as an inverted U-shape in our study.

Second, there is no doubt that the digital economy will enhance the technological content of products and optimize the structure of export products by promoting technological innovation and accumulating production factors such as knowledge and human capital. However, existing studies do not examine this aspect empirically. In addition, existing studies in this field ignore the correlation between mediating variables [12, 13], which can cause model estimation bias and lead to conclusions with deviations from reality. Therefore, we use a chain mediating effect model (CMEM) to reveal specific paths through which the digital economy affects urban ES.

Third, many earlier studies on ES are based on cross-country panel data [14], which cannot reflect the regional differences in ES within a country [15]. However, there exists regional differences in ES between developed cities in coastal China and some underdeveloped areas in western China [16]. With this in mind, this paper discusses the relationship between the digital economy and ES at the city level, considering regional heterogeneity. Our study shows that the impact of the digital economy on urban ES is regionally heterogeneous, with cities in the relatively underdeveloped western region having the greatest impact.

This paper provides a unique perspective on the impact of city-level digital economy development on the ES, enriches the research in related fields, and reveals how the digital economy affects the ES through human capital and technological innovation through mechanism analysis. This study not only promotes the understanding of digital economy and its economic effect in constructing new network infrastructure, but also provides valuable inspiration for cities to optimize export trade structure and achieve high-quality foreign trade development.

The rest of the study is organized as follows. Theoretical mechanism and research hypothesis section introduces the theoretical mechanism and research hypothesis; Model, Variable Setting, and Data Source section details the model setting process, variable selection description, and data sources. Subsequently, Empirical Results and Analysis section includes the empirical results, with an analysis of the results of the benchmark model, heterogeneity, quantile regression, and threshold effect test. Finally, Conclusions and Policy Implications section concludes the study and presents policy implications.

## Theoretical mechanism and research hypothesis

### The impact of digital economy development on urban export sophistication

A city’s ES can measure the upgrading of the structure of its export products and can be used as a representative indicator to measure export trade quality [16]. Previous studies have not directly analyzed the relationship between the development of the digital economy and urban ES. However, we can still draw on similar studies to form theoretical hypotheses. The wide application of digital technology and ICT has become a new source of comparative advantage in international trade [17, 18] In general, a high level of digital economy development is usually evident from a city’s well-developed internet infrastructure, popularization of internet users, strong internet integration and application capabilities, and favorable development environment. Good internet development is conducive to improving urban ES, primarily in the following aspects: first, it will optimize the urban industrial structure and promote the upgrading of the export product structure, and contribute to the achievement of sustainable development goals [19]. Empirical studies have demonstrated that internet development has optimized the product structure of trade, facilitating the increase of the technical complexity of urban export [20, 21]. Second, the development of cloud computing, big data analysis, and the Internet of Things has fostered a strong knowledge network, enabling enterprises to accelerate product innovation [22]. Third, the development of digital infrastructure promotes extensive information sharing and knowledge dissemination. Simultaneously, it effectively reduces the transaction cost of information acquisition and processing, promotes the integration of various innovation factors, and continuously supports export enterprises to upgrade products in the dynamic international competition [23, 24]. In summary, hypothesis 1 is as follows.

H1: The development of the digital economy has a significant and positive impact on urban ES.

### The impact mechanism of digital economy development on urban export sophistication

The impact mechanism of digital economy development on urban ES can be explained as follows.

#### Human capital effect

The increase in export technological complexity usually transforms traditional sectors into modern ones. A critical sign is the continuous accumulation of intelligence and human capital [25]. According to factor endowment theory, trade structure is mainly affected abundant human capital and other factors. The spatial agglomeration of specialized human capital will reshape the industrial division of labor’s spatial structure and optimize the industrial and trade structures. Studies have shown that the use of the internet and digital technology in the development of the digital economy has enriched the employment choices of workers and improved the allocation efficiency of human capital. Simultaneously, the efficiency of information matching is improved. Subsequently, the effect of “learning by doing” and human capital accumulation can be accelerated [26, 27]. Human capital is a “technology carrier.” Human capital accumulation is related to advanced technology and management experience to improve technological innovation ability. Therefore, human capital accumulation promotes ES [28, 29]. In summary, hypothesis 2 is as follows.

H2: Human capital is a mediating variable that promotes urban ES in the development of digital economy, that is, the existence of the “human capital effect.”

#### Technological innovation effect

Improving enterprise innovation level is crucial for improving the technological content and added value of products, improving trade products’ structure, and enhancing export competitiveness [30]. Studies have shown that the rapid development of the digital economy accelerated the transformation of innovation results. For example, big data can help enterprises integrate information and develop new products by visualizing decision-making paths [31]. At the industrial level, digital economy development promotes industrial digitization, which helps optimize the industry’s technological structure [32]. At the regional level, digital economy development can effectively improve regional total factor productivity and, thus, has an innovation-driven effect [33, 34]. For the urban development of the digital economy, the required knowledge and information as well as research and development (R&D) capital are more intensive. The digital economy plays a crucial role in allocating resources. It breaks the barriers of factor flow, promoting the optimization of urban trade structure and enhancing ES. Based on the abovementioned discussion, hypothesis 3 is as follows.

H3: Technological innovation is the mediating variable of digital economy development, which promotes the increase of ES; that is, the “technological innovation effect” exists.

## Model, variable setting, and data source

### Model construction

**Fig 1:**
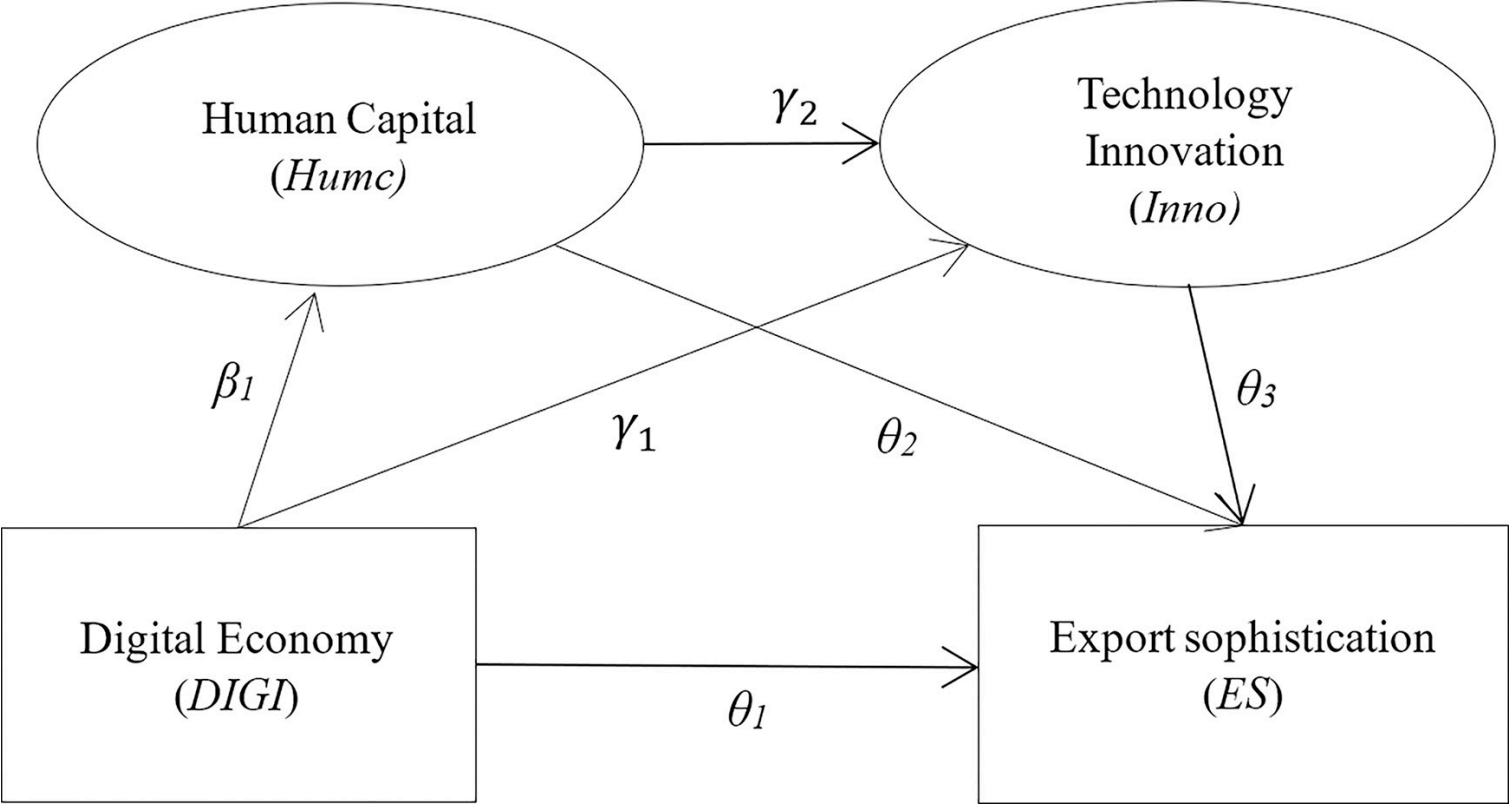
Chain mediation effects model.

The chained mediation effects model (CMEM) can be seen as a special case of directed acyclic graphs (DAG). Unlike the traditional mediation analysis [35], which has only one mediating variable, CMEM can, in principle, include more than one mediating variable and allow for correlations between mediating variables rather than independence. Fig 1 is a visualization of the CMEM. The two ellipses on the top show the mediating variables, and the explanatory and interpreted variables are shown in the two rectangles on the bottom side. In this paper, we construct a CMEM to test the mechanism of the digital economy’s impact on ES, which is divided into four steps.

Step 1: Use the dependent variable ES to regress the basic independent variable digital economy (DIGI).
Step 2: Use the mediating variable human capital (Humc) to regress the basic independent variable digital economy (DIGI).
Step 3: Use the mediating variable technological innovation (Inno) to simultaneously regress human capital (Humic) and the independent variable digital economy (DIGI).
Step 4: Use the dependent variable ES to simultaneously regress the two mediator variables and the primary independent variable.

By estimating this model, the total effect α_1_ of the digital economy (DIGI) on technological ES, the direct effect θ_1_, and the indirect effect measured by the mediation effect can be obtained. The steps above are represented by Eq (1) as follows:

$$\left\{ \begin{array}{l} {ES}_{it}=\alpha_{0}+\alpha_{1}{DIGI}_{it}+\alpha_{2}C_{it}+u_{i}+\nu_{t}+\varepsilon_{it}\\ {Humc}_{it}=\beta_{0}+\beta_{1}{DIGI}_{it}+\beta_{2}C_{it}+u_{i}+\nu_{t}+\varepsilon_{it}\\ {Inno}_{it}=\gamma_{0}+\gamma_{1}{DIGI}_{it}+\gamma_{2}{Humc}_{it}+\gamma_{3}C_{it}+u_{i}+\nu_{t}+\varepsilon_{it}\\ {ES}_{it}=\theta_{0}+\theta_{1}{DIGI}_{it}+\theta_{2}{Humc}_{it}+\theta_{3}{Inno}_{it}+\theta_{4}C_{it}+u_{i}+\nu_{t}+\varepsilon_{it} \end{array}\right.$$ (1)

In Eq (1), we log-transform the dependent variable ES. Subsequently, the digital economy (DIGI) coefficient is semi-elasticity, which refers to the percentage of the ES that changes when the digital economy changes by one unit. The subscript i of each variable represents the city, and t is the time. C_it_ indicates the control variable. *μ*_*i*_ represents the individual fixed effect. *ν*_*t*_ is the time fixed effect, and *ε*_*it*_ indicates the random disturbance term. The impact mechanism constructed by the model includes three paths, and the path coefficients are calculated from the coefficients of each variable in Eq (1).

Path 1: The development of the digital economy contributes to the technological sophistication of urban exports through the accumulation of human capital with a path coefficient of *β*_1_*θ*_2_.
Path 2: The development of the digital economy promotes the technological sophistication of urban exports by promoting technological innovation with a path coefficient of *γ*_1_*θ*_3_.
Path 3: The development of the digital economy promotes the technological sophistication of urban exports through the combined effect of human capital and technological innovation, that is, the chain of mediation effects, with a path coefficient of *β*_1_*γ*_2_*θ*_3_. (Fig 1) shows the CMEM constructed in this study.

### Variable selection

#### Explained variable

The measurement of the explanatory variable urban ES is derived from the widely quoted work of [36]. The idea is to weight a country’s per capita income by the ratio of a product’s share of a country’s total exports to the sum of that share for all exporting countries. Since labor productivity data reflecting technical indicators are difficult to obtain directly, the per capita gross domestic product of each country is often used as a proxy index. The calculation method of export technical complexity (*prody*_*h*_) at the product level in a specific year is shown in Eq (2), where *X*_*q*,*h*_ represents the export volume of product *h* from country *q*, *X*_*q*_ indicates the total export value of the product from country *q*, and *Y*_*q*_ represents the per capita gross national product of country *q*.


$${prody}_{h}=\sum_{q=1}^{n}\frac{\left(X_{q,h}/X_{q}\right)}{\sum_{q=1}^{n}\left(X_{q,h}/X_{q}\right)}\cdot Y_{q}$$ (2)

Referring to [37, 38] to measure the technical sophistication of city exports, we obtain the product-level data. Subsequently, we sum the same to produce the city-level data, using the product export value as the weight with which to obtain the data on the technical sophistication of the exports for each city, which is measured as per Eq (3).


$${ES}_{f}=\sum_{h=1}^{n}\frac{X_{f,h}}{X_{f}}\cdot {prody}_{h}$$ (3)

In Eq (3), the subscript *f* represents the city. *ES*_*f*_ is the ES at the city level. *X*_*f*,*h*_ is the export volume of *h* products in city *f*, and *X*_*f*_ is the total export volume of city *f*, $X_{f}=\sum_{h=1}^{n}X_{f,h}$.

#### Explanatory variable

The core explanatory variable of this study is the urban digital economy development level (DIGI). Since the relevant index of the urban digital economy has not been disclosed by the official government, we draw on the methods of [39] to measure the level of economic development. Thus, we measure internet development using five indicators: internet penetration, internet-related employment, internet-related output, mobile phone penetration, and development of digital finance (Table 1). The proxy variables of the first four aforementioned indicators are the number of internet broadband access users, information transmission and software services, the proportion of employed persons in urban units of the information technology service industry, the total telecom services per capita, and the number of mobile phone users per a population of 100 people. The proxy variable for development of digital finance is derived from the China Digital Finance Inclusive Index published by the Peking University Digital Finance Research Center. Finally, each indicator is standardized. The principal component analysis method gives weight to each indicator; subsequently, we reduce its dimension into a single indicator, which is each city’s digital economy development level (DIGI).

**Table 1:** Indicator system for measuring the development level of the digital economy in each province.

First-level indicator	Secondary indicators	Three-level indicator	Data Sources
**Digital economy development level**	Internet penetration	Internet broadband access users among 100 people	National Bureau of Statistics
	Internet-related employment	The proportion of employed persons in urban units in information transmission, software, and information service industries	
	Internet-related output	Total telecom services per capita	
	Mobile phone penetration	Number of mobile phone users among 100 people	
	Development of digital Finance	China Digital Finance Inclusive Index ^a^	Peking University Digital Finance Research Center

^a^ The Peking University Digital Financial Inclusion Index of China: https://en.idf.pku.edu.cn/docs/20190610145822397835.pdf

#### Mediating variable

This study, drawing on existing literature, examines the mediating variables of human capital (Humc) and technological innovation (Inno) [40]. Human capital accumulation is the key to the optimization of trade structure. Specifically, abundant human capital is convenient and enables its functions of “technology carrier” and “factor accumulation.” Urban human capital (Humc) is measured using the average number of college students per population of 10,000 people. For the other mediating variable, technological innovation (Inno), the number of patents granted per year in the city is selected to measure the city’s technological innovation level.

#### Control variable

Referring to the existing relevant research, we select three control variables at the city level: the first is the financial industry’s development level (Fina) [11]. Urban enterprises with a high financial industry development level have faceless financing constraints, which is conducive to upgrading export product structure. This variable is measured by the number of financial employees per population of 10,000 people in the city. The second is the transportation infrastructure (Tc) [40]. The convenience of transportation infrastructure directly affects the transaction cost and efficiency of export trade; therefore, it can potentially affect ES. Here, we measure it by the number of city road miles selected for this indicator. The third is the FDI (Fdi) [41], which impacts ES through effects such as technology spillover. This variable is measured based on the actual amount of foreign investment each city uses in the current year.

### Sample selection and data source

The data on the variables required to measure the digital economy are taken from the National Bureau of Statistics and the China Digital Inclusive Finance Index, as compiled by the Digital Finance Research Center of Peking University [42]. In this study, we select over 180 countries with export records from 2011 to 2017 as the research object to measure urban ES due to the data availability. Some national data are excluded owing to a lack of records in the sample interval. The two databases are matched according to the country’s name. Finally, the export data of more than 4,700 products from 135 countries are obtained. In addition, the calculation of ES data at the city level must also match that of the import and export statistics database of China Customs. Thus, the selection process of the sample cities is as follows: first, we eliminate and organize the loss of information samples in the database, sum up the monthly data to obtain the year data and filter and summarize the data according to the name of the export city. Second, we compare the urban export product data to the commodity code comparison table of the 1996 version, match the sample data, and then intercept the HS eight-digit code to the HS six-digit code. Finally, we classify and summarize the sample data according to the 2017 administrative division code published by the National Bureau of Statistics and select 281 cities as the research object. The required data are retrieved from the China Customs import and export statistics database, China research data service platform, World Bank database, United Nations Comtrade database, China Urban Statistics Yearbook, and various urban data disclosure information. The data used for this analysis can be found in S1 Data.

## Empirical results and analysis

### Descriptive statistical analysis

Table 2 shows the descriptive statistical results of the main variables. The mean value of urban ES is 49,596.230, the minimum value is 23783.7, and the maximum value is 75,352.400. The mean value of the digital economy development (DIGI) is 0, the minimum value is −2.027, and the maximum value is 5.122. These results indicate that the development of the digital economy in Chinese cities presents significant spatial differentiation.

**Table 2:** Descriptive statistics for primary variables.

Variable name	Variable meaning	Sample size	Mean	Standard deviation	Minimum value	Maximum value
***ES***	Export sophistication	1967	49596.230	8415.680	23783.7	75352.400
***DIGI***	Digital economy development level	1967	0.000	0.926	-2.027	5.122
***Inno***	Technological innovation	1967	0.450	1.030	0.0012	10.649
***Humc***	Human capital	1967	1.814	2.374	0.0174	12.704
***Fina***	Level of financial development	1967	43.980	39.422	5.992	476.670
***Tc***	Transport infrastructure	1967	1.905	2.412	0.052	19.015
***Fdi***	Foreign direct investment	1967	0.948	2.227	0.00003	30.826

(Fig 2) shows the spatial distribution of urban ES in 2007 and 2017, demonstrating that China’s urban ES has improved significantly and that the export trade structure has been optimized. However, clear spatial divergence characteristics are present. Cities in the eastern region have inherent geographical advantages, numerous special economic zones, and coastal cities with more developed high-tech industries. Thus, eastern urban ES is consistently in the national leading position. In contrast, urban ES is lower in the central region and lowest in the western region. (Fig 2) also shows that urban ES in Chinese cities present clear spatial agglomeration. Cities with high urban ES are primarily concentrated in the urban agglomerations of central and southern Liaoning, Beijing–Tianjin–Hebei, the Shandong Peninsula, the Yangtze River Delta, and the Pearl River Delta from north to south. The urban ES of Ningxia along the Yellow River, Xining–Lanzhou, the Guanzhong Plain, Central Guizhou, and central Yunnan is relatively low.

**Fig 2:**
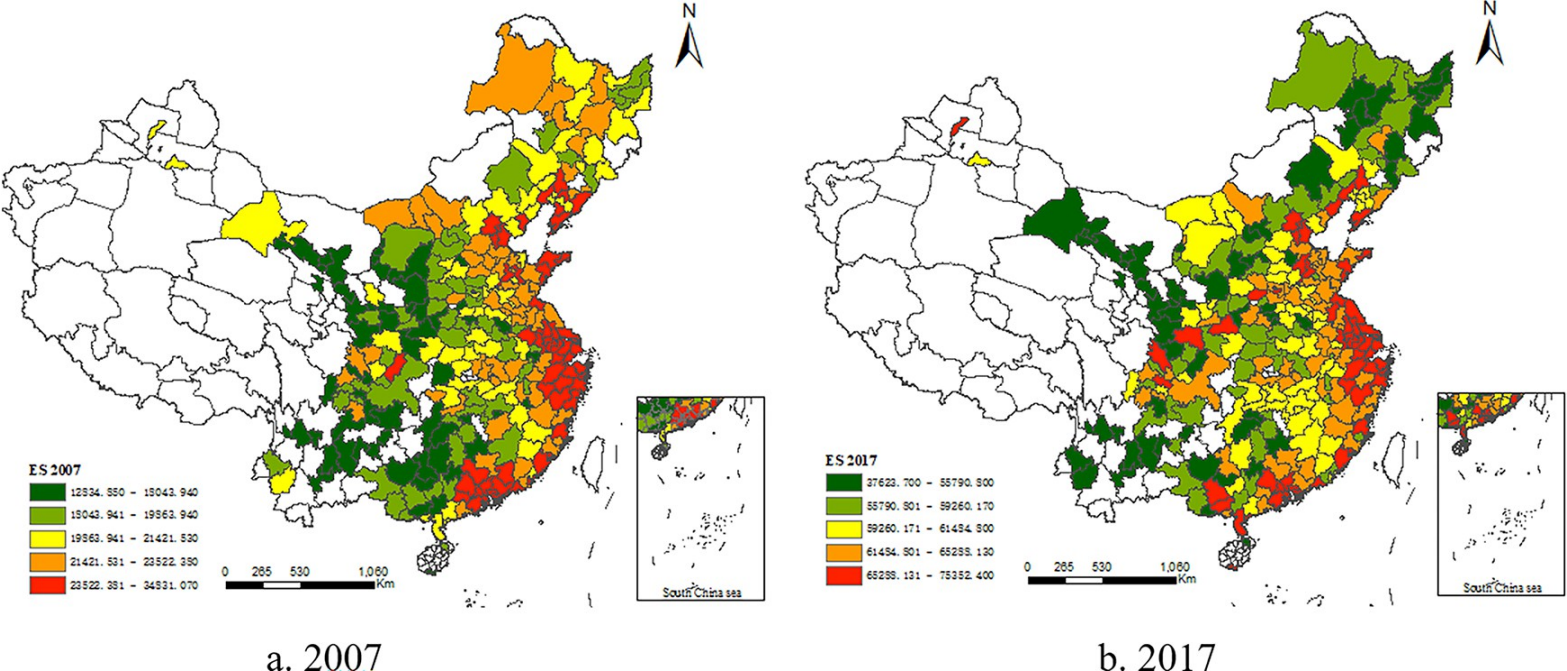
Spatial differentiation of urban exports sophistication (2007, 2017).

### Benchmark regression results

Before conducting the baseline regression, a Hausman test is conducted to examine the model’s applicability. The chi-square statistic is 129.36 with a p-value of 0. This rejects the hypothesis that the random disturbance term is not correlated with explanatory variables at the 1% significance level. Hence, the panel data suits the fixed effects (FE) model. The regression results are shown in Table 3. Column (1) lists the FE regression results, containing only the core explanatory variable digital economy development level (DIGI). Columns (2), (3) and (4) are the regression results based on column (1), with the gradual addition of control variables, namely, the level of financial development (Fina), transportation infrastructure (Tc), and FDI (Fdi). The results show that the explanatory variable coefficients are positive and significant at 1%, suggesting that digital economic development contributes significantly to urban ES, and hypothesis 1 is validated.

**Table 3:** Benchmark regression results of digital economy development on export sophistication.

Variable	(1)	(2)	(3)	(4)
	ES	ES	ES	ES
***DIGI***	0.210***	0.210***	0.211***	0.211***
	(71.832)	(72.216)	(73.102)	(73.067)
***res_Fina***		0.001***	0.001***	0.001***
		(3.830)	(4.272)	(4.158)
***res_Tc***			0.026***	0.025***
			(5.993)	(5.665)
***res_Fdi***				-0.003
				(-1.212)
***Constant***	10.797***	10.797***	10.797***	10.797***
	(5,982.264)	(6,006.225)	(6,068.340)	(6,069.022)
**Fixed effects**	YES	YES	YES	YES
**Observations**	1976	1976	1976	1976
***R*** ^***2***^	0.754	0.756	0.761	0.761

Note: The *t* values of the coefficients are in brackets. *, **, *** indicate significance at the 10%, 5% and 1% levels, respectively. The following tables are the same.

To solve the problem of variance expansion and coefficient reversal caused by multicollinearity, this study adopts the residualization method proposed by [43]. The advantage of this method is that it can effectively deal with multicollinearity problems and isolate the individual effects of explanatory variables. The variable res_Fina in Table 3 is the residual term from the regression of the financial development level (Fina) on digital economic development level (DIGI). res_Fdi is the residual term from the regression of FDI on financial development level (Fina) and digital economic development level (DIGI). res_Tc is the residual term from the regression of transportation infrastructure (Tc) on FDI, financial development level (Fina), and digital economy development level (DIGI). The residualization method can ensure the isolation of each explanatory variable and the independence of each explanatory variable in the regression model.

### Mechanism test

The mechanism test results are shown in Table 4. Column (1) presents the estimation results of the benchmark model. Column (2) lists the mediating variable human capital (Humc) to regress the core independent variable digital economy development (DIGI). The results show that the digital economy development (DIGI) coefficient is 0.142. It is significantly positive at the 1% level, indicating that digital economy development helps create an atmosphere and environment for innovation and promotes the accumulation of urban human capital. The results in column (3) come from regressing technological innovation (Inno) on both human capital (Humc) and digital economic development (DIGI), i.e., a test of chained multiple mediation effects. The coefficient of digital economic development (DIGI) is 0.153, which is significant at the 1% level. The coefficient of human capital (Humc) is 0.023. However, it is not significant, which indicates that a chain mediation effect does not exist. Column (4) presents the regression of the dependent variable ES on the core independent variable digital economic development (DIGI), the mediating variable human capital (Humc), and technological innovation (Inno). Their coefficients are all significant at the 1% level, suggesting that all three positively contribute to ES.

**Table 4:** Results of testing the mechanism of the development of the digital economy on the export sophistication.

Variables	(1)	(2)	(3)	(4)
	ES	Humc	Inno	ES
***DIGI***	0.211***	0.142***	0.153***	0.204***
	(73.068)	(9.571)	(13.002)	(65.962)
***Humc***			0.023	0.015***
			(1.241)	(3.206)
***Inno***				0.023***
				(3.73)
***res_Fina***	0.001***	-0.004***	-0.004***	0.001***
	(4.160)	(-4.122)	(-6.007)	(3.803)
***res_Tc***	-0.003	-0.141***	-0.086***	0.023***
	(-1.213)	(-5.944)	(-4.602)	(4.816)
***res_Fdi***	0.025***	-0.050***	-0.038***	-0.004
	(5.662)	(-3.507)	(-3.562)	(-1.493)
***Constant***	10.797***	1.814***	0.408***	10.759***
	(6,069.113)	(198.052)	(11.643)	(1,180.691)
**Fixed effects**	YES	YES	YES	YES
**Observations**	1967	1967	1967	1967
***R*** ^***2***^	0.761	0.085	0.138	0.762

**Fig 3:**
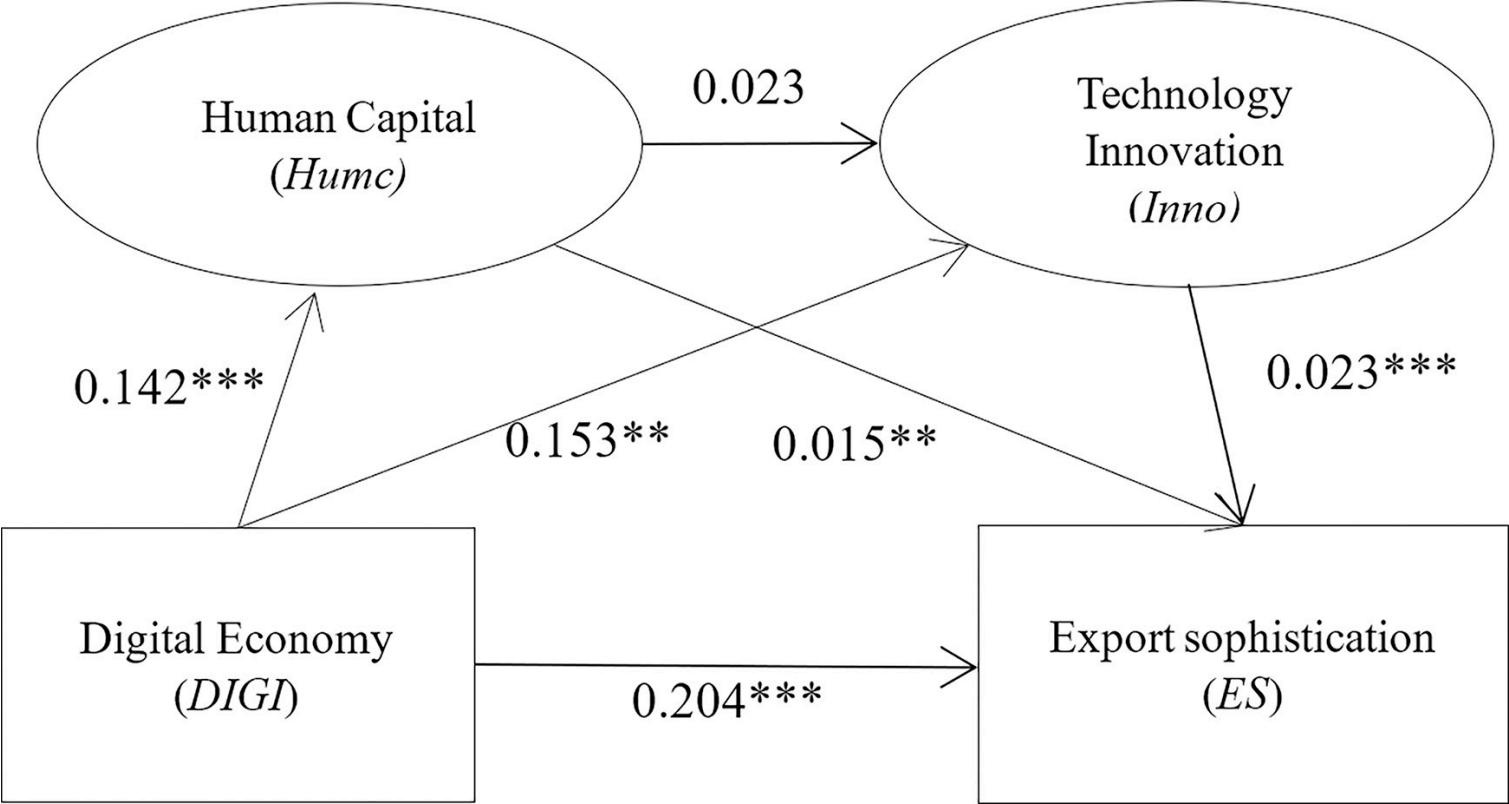
Test results of chain multiple mediation effect.

The aforementioned results reflect two possible channels for developing the digital economy to improve the technological sophistication of urban exports, namely, the “human capital effect” and the “technological innovation effect.” From the regression results in columns (2), (3) and (4) of Table 4, where each correlation coefficient is significantly nonzero, it can be estimated that the mediating effect is significant. The test results of the chain mediation effects are shown in (Fig 3). There are three mediation effects of digital economy development on urban ES. The coefficient of each path is the product of all coefficients on that path. Combined with the regression results in Table 5, the total effect of digital economy development on urban ES is *α*_1_ = 0.211, and the direct effect is *θ*_1_ = 0.204. The size of “human capital effect” for the indirect effect *β*_1_*θ*_2_ is 0.00213, “technological innovation effect” *γ*_1_*θ*_3_ is 0.003519, and “human capital–technical innovation common effect” *β*_1_*γ*_2_*θ*_3_ is 0.00007. The “technological innovation effect” is the largest among the three channels. In contrast, the role of “human capital–technological innovation common effect” is relatively small.

**Table 5:** Results of testing the mechanism of the development of the digital economy on the export sophistication.

Variables	(1)	(2)	(3)
	Eastern Region	Central Region	Western Region
***DIGI***	0.191***	0.219***	0.242***
	(34.004)	(43.028)	(31.347)
***res_Fina***	0.001***	0.001***	-0.000
	(3.122)	(3.507)	(-0.163)
***res_Tc***	0.006	0.015*	0.052***
	(0.742)	(1.937)	(4.260)
***res_Fdi***	-0.005	-0.004	-0.001
	(-0.904)	(-1.215)	(-0.057)
***Constant***	10.785***	10.814***	10.791***
	(3,136.175)	(2,306.44)	(1,313.089)
**Fixed effects**	YES	YES	YES
**Observations**	707	700	560
***R*** ^***2***^	0.711	0.798	0.727

Further, we test the significance of the mediation effect coefficient. For example, the Sobel test [44] statistic of “human capital effect” is $z={\hat{\beta }}_{1}{\hat{\theta }}_{2}/s_{\beta_{1}\theta_{2}}=3.043$, where $s_{\beta_{1}\theta_{2}}$ is the standard deviation of ${\hat{\beta }}_{1}{\hat{\theta }}_{2}$. The z value is far larger than the critical value of 1.96 at the 5% significance level. Therefore, the original hypothesis that H0: β_1_θ_2_ = 0 can be rejected, which shows that the “human capital effect” is significant. Similarly, combined with the data in Table 5, the corresponding z-values of the “technological innovation effect” and “human capital–technological innovation common effect” are 3.585 and 1.168, respectively. The corresponding P values are 0.00 and 0.121, respectively, indicating that the “joint effect of human capital and technological innovation” is insignificant. The chain mediation effect does not exist.

### Heterogeneity analysis

China is a vast country, and the natural and socio-economic environments vary greatly between North and South and East and West, so it is necessary to verify whether there is regional heterogeneity in the impact of the digital economy on ES. This study divides cities into eastern, central, and western regions according to their locations and analyzes the differential impact brought by different locations. The empirical results in Table 5 demonstrate that the promotion coefficients of the urban digital economy on ES are positive, indicating a promotion effect in the three regions. However, owing to the regional imbalance of urban economic construction and digital economic development, the eastern coastal areas with high economic development levels experience a higher promotion effect than the central and western regions. The impact of digital economic development on urban ES differs depending on the region. The western region’s urban digital economy development coefficient is 0.242 and is significant at the 1% level, higher than that of eastern and central China. The impact of the digital economy on ES might result in the phenomenon of diminishing marginal benefits. The impact on regions with lower levels of digital economy development is greater than that on regions with higher levels.

To verify this conjecture, we add the square term of digital economy development level (DIGI) into the regression model, as shown in Eq (4)

$${ES}_{it}=\lambda_{0}+\lambda_{1}{DIGI}_{it}+\lambda_{2}{{DIGI}_{it}}^{2}+\lambda_{3}C_{it}+u_{i}+\nu_{t}+\varepsilon_{it}$$ (4)

The partial derivative of the dependent variable to the core independent variable is $\frac{\partial ES}{\partial DIGI}=\lambda_{1}+2\lambda_{2}DIGI$. Suppose that the coefficient of the squared term is significant. Such a case indicates that the impact effects of digital economy development (DIGI) on ES vary, suggesting a threshold value for the variable DIGI. The results in Table 6 show that the coefficients of the squared terms at the overall and sub-regional levels are negatively significant at the 1% level, indicating an inverted U-shaped relationship between the development of the digital economy (DIGI) and ES. A “digital economy Kuznets curve” exists, so when the digital economy develops to a certain level, its influence on ES is suppressed. This is relatively consistent with the findings of [45].

**Table 6:** Regression results after adding the squared term.

	(1)	(2)	(3)	(4)
Variables	Integral	Eastern Region	Central Region	Western Region
***DIGI***	0.231***	0.230***	0.226***	0.239***
	(92.199)	(56.632)	(61.915)	(42.342)
***DIGIsq***	-0.035***	-0.033***	-0.039***	-0.044***
	(-27.538)	(-19.178)	(-15.497)	(-14.413)
***res_Fina***	0.001***	0.002***	0.000	0.003***
	(7.420)	(7.244)	(0.576)	(4.762)
***res_Tc***	0.041***	0.022***	0.039***	0.072***
	(11.179)	(3.974)	(6.554)	(7.737)
***res_Fdi***	-0.001	-0.003	-0.003	0.016
	(-0.296)	(-1.018)	(-1.057)	(1.370)
***Constant***	10.827***	10.821***	10.836***	10.853***
	(5,882.018)	(3,664.105)	(2,964.368)	(1,413.758)
**Fixed Effect**	YES	YES	YES	YES
**Observations**	1,967	707	700	560
***R*** ^***2***^	0.835	0.848	0.878	0.803

Note: *DIGIsq* denotes the squared term of *DIGI*

Furthermore, this study tests whether a threshold effect for the variable DIGI exists, using threshold regression to verify the side of the inverted U-shape on which the relationship between the current level of digital economy development (DIGI) and ES lies. The results of the threshold tests in Table 7 show that the third threshold value is not significant. The second threshold value is significant at both the overall and regional levels, showing a double threshold effect for the variable DIGI. As shown in Table 8, at the overall level and in the east, central, and west regions, the coefficients of the three intervals that correspond to the two thresholds show a decreasing trend. This confirms the diminishing marginal effect on the impact of the development level of the digital economy on ES. Thus, we can determine the current influence of the cities’ digital economy development in each region on the ES on the left side of the inverted U-shaped interval. The marginal effect is diminishing with the coefficients of the three intervals decreasing. We believe that the effect is currently in the upper half of the left-hand side of the inverted U-shaped interval.

**Table 7:** Threshold test results.

Region	Number of thresholds	Threshold value	F-value	P-value
	1	-0.008	765.550	0.000
**Integral**	2	0.009	72.940	0.000
	3	1.080	35.920	0.670
	1	0.023	339.210	0.000
**Eastern Region**	2	2.298	60.770	0.000
	3	0.844	10.140	0.79
	1	0.973	245.570	0.000
**Central Region**	2	1.080	49.500	0.000
	3	1.761	5.820	0.817
	1	-0.019	222.110	0.000
**Western Region**	2	1.786	20.910	0.023
	3	1.835	8.830	0.527

**Table 8:** Threshold regression results.

	(2)	(5)	(6)	(7)
Variables	Integral	Eastern Region	Central Region	Western Region
***Interval 1***	0.289***	0.284***	0.272***	0.307***
	(66.299)	(40.417)	(52.708)	(35.724)
***Interval 2***	0.185***	0.191***	0.198***	0.171***
	(24.928)	(14.127)	(21.882)	(17.304)
***Interval 3***	0.147***	0.155***	0.146***	0.099**
	(31.762)	(21.655)	(18.139)	(2.108)
***res_Fina***	0.001***	0.002***	0.001***	0.003***
	(8.116)	(8.145)	(2.869)	(5.088)
***res_Tc***	0.042***	0.023***	0.039***	0.074***
	(11.528)	(4.155)	(6.573)	(7.874)
***res_Fdi***	-0.001	-0.003	-0.003	0.017
	(-0.291)	(-0.898)	(-1.007)	(1.456)
***Constant***	10.844***	10.838***	10.840***	10.867***
	(3,605.286)	(2,272.378)	(2,719.943)	(1,286.398)
**Fixed Effect**	YES	YES	YES	YES
**Observations**	1,967	707	700	560
***R2***	0.837	0.850	0.883	0.805

Note: *Interval 1* indicates the left interval of the first threshold. *Interval 2* indicates the proper interval of the first threshold and the left interval of the second threshold. *Interval 3* indicates the right interval of the second threshold.

### Robustness tests

#### Instrumental variable method

The Hausman test results showed that the null hypothesis (H_0_) that “all explanatory variables are exogenous” was rejected. Thus, endogeneity problems may exist owing to omitted variables and other issues. The instrumental variable (IV) method is selected for the endogeneity. The IV for the digital economic development (DIGI) is identified as the number of fixed telephone calls per a population of 100 people in cities in 2000, with reference to [46] study. The IVs are selected for the following reasons: first, cities that were among the first to develop landline telephones may also be ahead of other cities in terms of their digital economy development, and the indicator satisfies the correlation. Second, the number of landline telephones per 100 people in each city historically has no impact on the city’s current ES; therefore, the indicator simultaneously satisfies the exogeneity requirement. Further, an IV test is used to verify whether using the number of fixed telephone calls per 100 people in 2000 is reasonable as an IV for the development of the digital economy. The results show that the *F* statistic of the first stage in the two-stage least squares (2SLS) regression is 16.906, which is far higher than 10 and significantly indicates that the choice of IV is reasonable. Column (1) of Table 9 shows the regression results using the IV method. The coefficient of digital economic development (DIGI) remains significantly positive.

**Table 9:** Robustness tests.

Variables	(1)	(2)	(3)
	IV method	2SLS method	Replace DIGI
***DIGI***	0.214***	0.230***	0.017***
	(7.389)	(54.535)	(5.545)
***res_Fina***	0.001***	0.001***	-0.000
	(3.417)	(5.258)	(-0.594)
***res_Tc***	0.025***	0.034***	0.015
	(5.423)	(6.796)	(1.448)
***res_Fdi***	-0.003	0.001	-0.005
	(-1.161)	(0.339)	(-1.381)
***Constant***	10.797***		10.955***
	(6,067.317)		(3,409.814)
**Fixed effects**	YES	YES	YES
**Observations**	1967	1686	562
***R* ^*2*^**	0.761	0.667	0.119

#### Explanatory variable lag for three periods

We allow the urban digital economy development level (DIGI) to lag for two periods. The *F* statistic is 3395.324, considerably higher than 10, which is the empirical rule, and it significantly rejects the null hypothesis. Thus, it is reasonable to take DIGI’s two-period lag as DIGI’s IV. The results of the 2SLS regression are shown in column (2) of Table 9. The impact of the digital economy on urban export complexity remains positive and statistically significant at the 1% level, indicating that the regression result is reliable.

#### Substitution of explanatory variable indicators

The measured index of digital economy development (DIGI) is replaced for re-estimation. The Urban Internet Development Index, which the Tencent Research Institute published, is used as the substitute index for digital economy development to verify the robustness of the results of this research. The results are reported in column (3) of Table 9, where the coefficient of digital economic development (DIGI) remains significantly positive at the 1% level. This supports the conclusion that DIGI has contributed to increasing the urban ES.

To verify the robustness of the results of the regional heterogeneity analysis, we simultaneously generate regression results for the five quartiles of 1%, 25%, 50%, 75%, and 90% (Table 10). This verification indicates that the impact of digital economy development (DIGI) on ES decreases from the western region to the eastern region, verifying the robustness of the heterogeneity analysis results in Table 5. It also reaffirms the diminishing marginal effect of digital economy development (DIGI) on ES.

**Table 10:** Quantile regression results.

	(1)	(2)	(3)	(4)	(5)
Region	0.1 Quantile	0.25 Quantile	0.5 Quantile	0.75 Quantile	0.9 Quantile
**Eastern Region**	0.186***	0.188***	0.191***	0.194***	0.195***
	(10.685)	(15.085)	(21.922)	(17.596)	(13.923)
**Central Region**	0.222***	0.221***	0.219***	0.218***	0.217***
	(19.676)	(27.307)	(36.752)	(27.086)	(20.946)
**Western Region**	0.224***	0.233***	0.243***	0.251***	0.257***
	(12.010)	(18.757)	(29.766)	(25.619)	(19.446)

https://search.crossref.org/?q=Drivers+of+export+upgrading&from_ui=yes

## Conclusions and policy implications

### Conclusions

The present research uses the panel data of 281 cities in China from 2007 to 2017 to measure the statistical index of ES of cities. Subsequently, the research delves into the impact of digital economy development on the ES of cities and its mechanism of action, obtaining the following main findings.

First, China’s urban ES shows spatial heterogeneity, with cities in the eastern region consistently leading the country in ES. Simultaneously, the city ES shows clear spatial clustering. Cities with high ES are concentrated in the eastern and southern coastal areas, while cities with low ES focus on the northeast, northwest and southwest regions.

Second, the digital economy’s development optimizes the product structure of urban exports. It significantly contributes to urban ES. To be more specific, there are two main influence paths. One promotes urban ES by accumulating human capital; the other stimulates technological innovation and leads to urban ES. Among them, the impact of the “technological innovation effect” is the most obvious.

Third, regional differences exist in the impact of digital economy development on the technological sophistication of urban exports. Compared to the eastern and central regions, digital economy development in cities in the western region has a greater increase in ES because of the diminishing marginal effect. In addition, we verify that the relationship between digital economy development and urban ES is positively correlated, and the marginal effect gradually decreases.

### Policy implications

This study provides valuable inspiration for promoting the digital economy at the city level, optimizing the export structure of cities, and achieving high-quality trade development. First, the construction of new digital economy infrastructure and the smart city should be promoted in the future, and the construction of the Urban Big Data Centre, cloud computing, a 5G network, and other infrastructure should be strengthened. Through an in-depth integration of the digital economy and various industries, the structure of urban export should be optimized, and the development quality of urban export trade should be improved.

Second, the study emphasizes the role of the “human capital effect” and “technology innovation effect” in developing the urban digital economy to increase ES. On the one hand, give full play to the role of digital economy in accelerating the accumulation of human capital, improving the attractiveness of cities to high-level and skilled workers through the integration of resources of digital economy, and attracting and cultivating more technical talents with diversified measures.

On the other hand, policymakers can further stimulate the R&D motivation of high-technology product exporters through research and innovation investment and policy preferences. Enterprises should take appropriate risk-averse measures and continue strengthening the digital development of trade against increased uncertainty in the current international economic environment. Third, given the heterogeneity of the impact of digital economy development on urban ES, accelerating the digital economic construction and digital economy in cities in the central and western regions is essential.

### Limitations and future research directions

The limitations of this paper include the following aspects. Because of data availability, this paper is not obtaining the latest data. In the future, the data should be updated to expand the sample size; moreover, microenterprise data could be applied in the future to verify the impact of the digital economy on ES and its mechanisms. In terms of methodology, this study does not fully consider the impact of spatial correlation and spatial heterogeneity. In fact, the impact coefficients may differ significantly across cities. Therefore, future studies should consider optimizing the methodology proposed in the literature [47, 48]. In addition, the CMEM can also incorporate more mediating variables in order to explore the mechanism of action between the digital economy and ES in more depth. However, it is worth noting that the complexity of the model will increase accordingly with each additional mediating variable. For example, when the mediating variables are increased from two to three, the potential influence paths increase from four to 16. In addition, the interrelationships among the mediating variables need to be examined at the same time, especially the issue of endogeneity due to mutual causation. These are important aspects that need to be further improved and explored in depth in future studies.

## Supporting information

S1 Data (DTA)
